# Associations between the trajectories of free sugar intake and cardiometabolic risk markers during childhood: generation XXI birth cohort

**DOI:** 10.1007/s00394-025-03688-9

**Published:** 2025-05-30

**Authors:** Sofia Sosa, Milton Severo, Catarina Campos Silva, Carla Lopes

**Affiliations:** 1 EPIUnit – Institute of Public Health, University of Porto, Porto, Portugal; 2 Laboratory for Integrative and Translational Research in Population Health (ITR), Porto, Portugal; 3 Department of Public Health and Forensic Sciences, and Medical School, Faculty of Medicine, University of Porto, Porto, Portugal; 4 Insitute of Biomedical Science Abel Salazar, University of Porto, Porto, Portugal; 5 Institute of Public Health, University of Porto, Rua das Taipas 135, Porto, 4050-600 Portugal

**Keywords:** Free sugar intake, Birth cohort, Free sugar trajectories, Cardiometabolic risk markers, Childhood

## Abstract

**Introduction:**

Free sugar (FS) intake is linked to obesity, but most evidence is cross-sectional. Few studies have explored early-life FS intake trajectories with later cardiometabolic risk markers (CRM).

**Aim:**

To estimate associations between the trajectories of FS intake and CRM during childhood using a longitudinal and cross-sectional analysis and two dietary assessment methods.

**Methodology:**

Participants are from the G21 cohort evaluated at 4, 7, 10, and 13y. Dietary intake was assessed through food diaries and food frequency questionnaries (FFQ). FS was measured using a 10-step adapted methodology based on the WHO definition. CRM analyzed included BMI, waist circumference (WC), HOMA-IR, HDL, triglycerides, and systolic blood pressure. Linear mixed-effect models were used to estimate cross-sectional and longitudinal associations between FS trajectories, and CRM. Two regression models were considered: model 1 (crude) and model 2 (adjusted for parity, mothers’ education, mothers’ weight pre-pregnancy, breastfeeding, physical activity, weight for gestational age, and energy intake).

**Results:**

Considering data from food diaries and after adjustment, the longitudinal analysis presented positive significant associations between FS intake(/10 g) and CRM, particularly within adiposity variables: BMI (β = 0.044, 95%CI = 0.000;0.088), WC (β = 0.240 95%CI = 0.093;0.386) and HOMA (β = 0.015, 95%CI = 0.003;0.028). In contrast, cross-sectional analyses showed negative associations for the same variables. When using data from FFQ similar results were observed as in diaries. A sensitivity analysis only in plausible reporters supported the previous results in both epidemiological approaches.

**Conclusion:**

This study supports the adverse effects of FS on CRM during childhood, independently of the dietary assessment method. Longitudinal approaches are relevant to obtaining accurate associations.

## Introduction

In the last decades, diets worldwide have become sweeter, with a notable increase in the intake of Free Sugars (FS) [1], which allude to monosaccharides and disaccharides added to food/drinks as well as natural ones that already exist in some food/drinks, e.g., fruit juices, honey [2]. Nevertheless, the intake of FS differs by age, setting, and country, although it is still higher among children [3].

FS has acknowledged attention to researchers, the public health perspective, and policymakers in the last few years. Not only because it has been linked with weight gain and poorer dietary qualities [2] but also because of the association with a higher risk of chronic diseases such as diabetes mellitus (DM) [4–7] and cardiovascular diseases (CVD) [8–10]. Emerging evidence has shown that cardiometabolic risk can begin in early life and continue to adulthood [11–13]. Furthermore, it has been confirmed that young children with severe obesity have a much higher risk of developing cardiometabolic complications [12, 14–16]. Some complications associated with body fat accumulation are hyperinsulinemia, insulin resistance, prediabetes, and impaired glucose tolerance (which is a strong predictor for the development of T2DM in youth obese) [12].

The World Health Organization (WHO) has recommended a ≤ 10% of the total energy intake (TEI) from free sugars and an additional reduction below 5% daily (25 g or six teaspoons) to observe health benefits [2]. From a global perspective, out of 25 countries, children’s total sugar intake (% total energy intake) ranged from 8% in China to 28.6% in the Netherlands, and in adolescents, it ranged from 15.4% in Italian boys to 30% in Slovenian girls [17]. Additionally, FS intake in children between 3 months and 10 years (y) ranged from 9.1% in Portuguese boys to 19.5% in Dutch girls from 4-8y. In adolescents, FS intake ranged from 9.1% in Portuguese (10-17y) and 17.9% in Dutch boys (9-13y) [17]. In Portugal, data from the National Food, Nutrition, and Physical Activity Survey (IAN-AF 2015–2016) showed that the mean total sugar intake in children is 22.7% and in adolescents 20.2%, and from FS is 11% [18]. In addition, the intake of sugar-sweetened beverages (SSBs) in children and adolescents (3-19y) from 185 countries increased by 23% from 1990 to 2018 [19].

Some studies [20] and a systematic review (SR) in children and adults [21] have reinforced the connection between sugar intake, particularly from SSBs, and weight gain. It also has been seen that children between 5 and 12 years of age consuming SSBs have an association with increased BMI values and a higher risk of being overweight/obese [9]. Also, research supports the positive association of added sugar (AS) with weight gain [22, 23]. However, most studies available only countFS from SSBs, not considering solid foods. To our knowledge, the overall FS intake (solid and liquid) concerning cardiometabolic risk markers has yet to be studied in children. Most studies have only focused on adults, and whether there is a relation in children is unclear or unknown.

Moreover, when assessing dietary intake, there can be different methods, each with its strengths and limitations, and the choice between them could influence the accuracy and reliability of the dietary data. In the current study, the dietary assessment was performed through food diaries and food frequency questionnaires (FFQ), allowing a comparison of the influence of the assessment methods in the associations.

The epidemiological approach used in studies can also gather evidence and draw different conclusions. Most studies assume a cross-sectional approach, often associated with reverse causality, particularly in sugars and sources of FS. Longitudinal studies provide crucial insights into how dietary habits, such as FS intake, can affect cardiometabolic health over time. There is little evidence of longitudinal relation between FS and cardiometabolic risk markers, particularly in young populations.

Therefore, this paper aims to estimate the association between the trajectories of free sugar intake and cardiometabolic risk markers during childhood using both longitudinal and cross-sectional analysis and two different dietary assessment methods.

## Methods

### Study design and study population

This study was embedded within the ongoing prospective population-based birth cohort Generation XXI (G21) established in Porto, Portugal, previously described [24, 25]. All newborn babies from five level III public maternity units in the metropolitan area of Porto born alive with ≥ 24 weeks or more of gestation between 2005 and 2006 were invited to participate. At baseline, 8647 newborns enrolled with a participation proportion of 91%. Baseline data was collected in the first 72 h after the delivery and during the hospitalization through face-to-face interviews. Data collected included demographics, social conditions, and lifestyles (diet, physical activity, sleeping habits, and medical care). All children were invited for follow-ups at ages four, seven, ten, and thirteen. The participation proportion was 86%, 80%, 76%, and 54% (interrupted by covid-10 pandemic), respectively. The eighteen-year-old follow-up is currently ongoing. All follow-up evaluations included objective measures of body composition, blood pressure, and blood analysis.

For the current study, inclusion criteria incorporated data on dietary intake through food diaries and FFQ at 4, 7, 10, and 13 years of age and blood analysis to collect some cardiometabolic risk markers. The exclusion criteria comprised, if twins, one was randomly excluded (n.149), infants with characteristics that could influence dietary intake like cerebral palsy, other congenital anomalies, and celiac disease (n. 55).

The final sample using FFQ questionnaires comprised 5554 children at four years of age, 5569 at seven years of age, 5097 at ten years of age, and 4347 at thirteen years of age. For food diaries, 2438 children at four years of age, 3494 at seven years of age, 2788 at ten years of age, and 2760 at thirteen years of age.

### Dietary intake

Dietary intake data was assessed through food diaries and FFQ. Food diaries covered three days, two weekdays, and one weekend day, which has been previously described [26]. Briefly, at 4, 7, and 10 years of age, children’s parents or, in the absence of them, their primary caregiver was asked to describe all foods and beverages consumed (as detailed as possible, including commercial brands, if applicable) and the amount in grams, units, or household measures. At 13 years of age, adolescents self-reported their diaries and asked their parents for help when needed. Written instructions were provided to assist with the fulfillment of the diaries, including an example of a complete food diary and photos of household measurements to help with food quantification.

The FFQ was applied to the parents or child’s primary caregiver at 4, 7, and 10 years; at 13 years, FFQ was self-reported by the adolescent with the support of the interviewers. At all waves, it was applied by a trained staff member, which covered the consumption of each food item in the previous six months and only at thirteen in the last twelve months. The FFQ was previously validated and calibrated in a sub-sample from G21 [27]. The total items used in the FFQ at four years were 35, seven years 38, ten years 41, and thirteen years 39. There was a nine-point frequency scale for the response options ranging from “never” to “4 times or more per day”.

After handling the dietary data in all waves, instructed nutritionists computerized the food diaries and FFQ using the methodology of the software eAT24 (Electronic Assessment Tool for 24-hour recall) from the National Food, Nutrition and Physical Activity Survey of the Portuguese Population (IAN-AF 2015–2016) [28]. Food items were classified according to the FoodEx2 system [29]; complex food or recipes were broken down into single ingredients as recommended by the EU Menu methodology [30]. Total energy and macronutrients were estimated based on the Portuguese Food Composition Table at PortFir (http://portfir.insa.pt), updated with the European Food Information Resource (EuroFIR) [31] data.

### Free sugars

The database had total sugar (TS) values in every individual food item. All recipes were broken down into single ingredients, and all TS estimations from individual recipes were based on the quantity of each single ingredient.

Free sugar and added sugar per item have been previously estimated in a dietary dataset [18]. A ten-step methodology was followed to measure FS and AS [32]. *Step 1*: foods with zero grams of total sugars allocate 0 g of AS *Step 2*: for unprocessed foods or slightly processed foods with no AS, allocate 0 g of AS. *Step 3*: foods with a slight amount of naturally occurring sugars allocate 100% of total sugars as AS. *Step 4*: a calculation constructed on the standard recipe used in the food composition database, using all ingredients following the previous steps. *Step 5*: is a calculated formula that is based on the comparison with values from unsweetened variety. *Step 10*: assign 50% of total sugars as AS. From the original proposed methodology, steps 6–9 were not used.

### Anthropometric data

During all follow-up assessments, trained professionals collected weight and height data. Weight was measured with no shoes and in light clothing to the nearest 0.1 kg using a scale (TANITA^®^ model TBF 300). A stadiometer (SECA 206 Hamburg, Germany^®^) measured height to the nearest 0.1 cm. BMI was computed, and BMI standard deviation (SD) scores were calculated following the WHO growth charts [33]; obesity prevalence was obtained according to obesity status classified according to WHO subsequent categories: thinness < 2 standard deviation (SD), normal weight ≤ + 1 SD, and overweight/obesity > 1 SD [34]. Waist circumference (WC) was measured to the nearest 0.1 cm; the child was standing, measured at the umbilicus level, with the abdomen relaxed and arms at the sides and feet positioned together.

### Cardiometabolic risk markers

Blood samples were obtained by trained nurses in our research department after an overnight fasting. A routine analysis was performed, including hemogram, lipid profile, insulin, and glucose. Triglycerides (TG) and high-density lipoprotein-cholesterol (HDL) were measured by the enzymatic colorimetric assay [35] using the Olympus AU5400 equipment (Beckman-Coulter, Brea, CA, USA). The homeostatic model assessment-insulin resistance (HOMA-IR) was computed as “fasting glucose (mg/dL) x fasting insulin (mU/mL)/405” [36]. From this, glucose was measured using an ultraviolet enzymatic assay (hexokinase method), and insulin levels were determined using an electrochemiluminescence immunoassay.

Systolic blood pressure (SBP) was measured twice using an automatic sphygmomanometer (Medel^®^ ELITE, S.Polo de Torrile, Italy) at the right brachial artery, with at least 5-minute intervals. A third measurement was implemented if a disparity was more significant than five mmHg between the measurements, and the mean between the closest values was considered. SBP z-scores were obtained based on the absolute values of height, age, and sex of G21 data.

### Covariates

Regarding the mothers’ characteristics at baseline, the variables used were education level, age, weight pre-pregnancy, and parity, which were all collected through face-to-face interviews.

Education was measured according to the total years of formal and completed learning years classified by the International Standard Classification of Education 2011 classes [37]: 9 or fewer years of education being the lowest, intermediate less or equal to 12 years, and 13 or more the highest. Weight presented in kg, BMI pre-pregnancy was calculated by BMI (kg/m^2^), parity (the number of times a woman has given birth to a baby of viable gestation or fetal weight, regardless of the birth outcome) given in number.

Regarding children, the information gathered was birthweight, weight for gestational age, breastfeeding, and physical activity. Birthweight was given in kg at birth, and weight for gestational age was computed as described in the statistical analysis section. Breastfeeding (asked at four years of age) and physical activity (asked at all waves) were yes/no questions.

### Statistical analysis

Descriptive statistics were used to summarize the participants’ general characteristics. For continuous variables, either the mean and standard deviation (SD) or the median and interquartile range (IQR) were reported, depending on the distribution. Categorical variables were described using absolute and relative frequencies.

A linear mixed-effects model was used to estimate the FS intake between the cross-sectional and longitudinal associations. Model 1 (M1) represents the crude, and model 2 (M2) the adjusted. It was adjusted for parity, mothers’ education, mothers’ weight before pregnancy, breastfeeding, physical activity, weight for gestational age, and energy intake.

A generalized additive model with integrated smoothness estimation function was used to compute the weight for the gestational age of the children. This model uses the non-linear association between the gestational age (adjusted by sex) and the weight at birth. Afterward, a new variable was created, considering the observed birth weight minus the predicted birth weight, to determine if the observed weight at birth was above or below the expected gestational age.

A sensitivity analysis is presented to assess the trajectories of FS intake, and the associations are only considered in plausible reporters. For the food diaries, misreporting of the energy intake was estimated using the Goldberg methodology [38], which Black [39] adapted later. The ratio of reported energy intake to predict basal metabolic rate (pBMR) was calculated by Schofield equations [40] considering fixed categories for physical activity (PAL = 1.6) and multiplied by a factor. This factor includes a correction for sample size, for the number of reported days (*n* = 3), and for the variation of energy intake (23%), pBMR (8.5%), and PAL (15%). Participants are defined as under, plausible, or over-reporters whether the individual ratio of EI: pBMR is below, within, or above the 95% confidence limits calculated, respectively, corresponding to 2 standard deviations (SD).

For the FFQ, misreporting was assessed by calculating the ratio between the reported total energy intake (TEI) through the FFQ and the estimated energy requirement (EER) [41, 42]. EER was estimated using sex and age-specific equations [43], considering the low active physical activity, exact age, weight, and height. Participants were categorized as plausible reporters, under-reporters, or over-reporters of TEI using the ± 1SD cut-off for the ratio TEI/EER [41, 42].

The cross-sectional and longitudinal associations of FS are given by 10 g.

All statistical analyses were performed using R version 4.4.0 using the package nlme.

Associations were presented with coefficients (β) and respective 95% confidence intervals (95% CI). A significance level of 5% was considered.

## Results

Table 1 presents the general characteristics of the participants and their mothers. The study includes 8443 children at baseline. The children’s mothers had a median age of 29.0 years (IQR = 25.0; 33.0), 59% of the mothers declared it was their first child, the median years of the mother’s education was 10.0 years (IQR = 7.0; 12.0), the mother’s median pre-pregnancy weight was 60 kg (IQR = 54; 67), 93% (6741) of the mother breastfed their children, the median weight at birth was 3.200 kg (IQR = 2,900; 3,500).

**Table 1:** General characteristics of the participants at baseline

*N*		8443
**Mother’s age** median (IQR)		29.0 (25.0; 33.0)
Missing		6
**Parity n(%)**	0	4,846 (59%)
	1–2	3261(39%)
	> 3	161 (2%)
Missing		175
**Mother’s education years** median (IQR)		10.0 (7.0; 12.0)
Missing		55
**Mother’s weight before pregnancy kg median (IQR)**		60 (54; 67)
Missing		179
**Mother’s weight before pregnancy — BMI n(%)**	< 25 underweight and normal weight	5365 (64.2%)
	25–29.9 overweight	1681 (20%)
	> 30 obese	684 (8.1%)
Missing		1224
**Breastfeeding n(%)**	Never	474 (6.6%)
	Yes	6741 (93%)
	Doesn’t know	4 (< 0.1%)
Missing		1224
**Breastfeeding duration n(%)**	Never	587 (8.3%)
	< 6 months	3524 (50%)
	≥ 6 months	2933 (42%)
Missing		1603
**Weight at birth kg** median (IQR)		3,200 (2,900; 3,500)
Missing		1

Moreover, Table 2 comprises a general description of the dietary exposures, physical activity, and cardiometabolic risk markers by wave. It was observed that energy (kcal) from diaries or FFQ intake were similar at all four waves. Still, diaries have a marginally higher energy intake compared to FFQ (1602 vs. 1444, 1766 vs. 1687, 1904 vs. 1855, and 1840 vs. 1791, respectively). The energy intake increases as children grow older, although there is a slight decrease at 13 years. It was also observed that the energy intake (%E) from FS varies from 9.2 to 10.6%E in diaries and 9.0 to 11.6%E in FFQ. The intake increases as children grow, with a pick in FFQ at 7y (11.6%E), and in diaries, it was observed at 10y (10.6%E); nevertheless, a minor decrease is appreciated at 13 years in both approaches. In physical activity, there are variations at all waves (4y 70%, 7y 55%, 10y 85%, and 13y 61%) from the participants who answered yes to structured leisure physical activity or practicing sports after school. For the other variables covering the cardiometabolic risk markers, an increase is perceived as children grow older, as expected.

**Table 2:** General description of the dietary exposures, physical activity, and cardiometabolic risk markers by wave

		4 years	7 years	10 years	13 years
Diaries Energy (kcal)	N	2437	3493	2788	2759
	Mean (SD)	1602 (293)	1766 (308)	1904 (385)	1841 (434)
FFQ Energy (kcal)	N	5554	5571	5096	4348
	Mean (SD)	1444 (295)	1686 (393)	1855 (416)	1792 (593)
Diaries Free sugar (g)	N	2437	3493	2788	2759
	Mean (SD)	36.9 (17.7)	46.8 (22.2)	50.7 (25.3)	48.5 (29.4)
	%E	9.2%	10.6%	10.6%	10.5%
FFQ Free sugar (g)	N	5554	5571	5097	4348
	Mean (SD)	32.7 (18.8)	49.0 (27.4)	52.7 (27.7)	50.7 (34.7)
	%E	9.0%	11.6%	11.3%	11.3%
Physical Activity – % yes	N	7205	4827	5674	6222
	n (%)	5019 (70%)	2669 (55%)	4837 (85%)	3823 (61%)
BMI (kg/m^2^)	N	5791	5689	5225	4514
	Mean (SD)	16.3 (1.8)	17.1 (2.5)	18.8 (3.4)	20.7 (3.8)
Waist circumference (cm)	N	5775	5681	5216	4505
	Mean (SD)	52.8 (4.5)	59.2 (6.9)	68.1 (9.8)	74.3 (10.2)
Glucose (mg/dL)	N	1497	4470	3764	3693
	Mean (SD)	79.0 (8.3)	83.2 (7.6)	87.2 (7.8)	89.1 (9.2)
Insulin (mU/mL)	N	1493	3929	3755	3650
	Mean (SD)	5.1 (5.8)	5.6 (4.2)	9.5 (6.3)	13.0 (8.5)
HDL (mg/dL)	N	1497	4470	3762	3694
	Mean (SD)	49.8 (10.2)	56.0 (10.7)	55.5 (10.6)	51.4 (9.8)
Triglycerides (ml)	N	1497	4470	3763	3694
	Mean (SD)	68.8 (30.4)	68.8 (35.8)	67.2 (33.9)	66.6 (30.3)
Z-score SBP	N	4607	5666	5209	4496
	Mean (SD)	0.006 (1.003)	-0.002(1.001)	0.011 (0.989)	-0.020 (1.004)

### Cross-sectional and longitudinal associations

Table 3 analyzes the cross-sectional and longitudinal associations between FS intake (by 10 g) and cardiometabolic risk markers using diaries and FFQ. The table presents the crude model (Model 1- M1) and the adjusted (Model 2- M2).

**Table 3:** Cross-sectional and longitudinal associations of FS (by 10 g) with cardiometabolic risk markers

	BMI (kg/m^2^)		WC (cm)		HOMA- IR		HDL (mg/dL)		TG (ml)		Z-score SBP	
	M1	M2	M1	M2	M1	M2	M1	M2	M1	M2	M1	M2
**FOOD DIARIES**												
FS Cross-section	-0.046 **(-0.076; − 0.015)**	-0.033 (-0.071; 0.005)	-0.191 **(-0.294; -0.088)**	-0.188 **(-0.315; -0.060)**	-0.013 **(-0.022; -0.005)**	-0.005 (-0.016; 0.005)	0.103 (-0.001; 0.208)	0.073 (-0.062; 0.209)	0.215 (-0.144; 0.576)	0.720 **(0.249; 1.191)**	0.010 **(0.000; 0.020)**	0.008 (-0.005; 0.021)
FS Longitudinal	0.221 **(0.186; 0.255)**	0.044 **(0.000; 0.088)**	0.891 **(0.773; 1.009)**	0.240 **(0.093; 0.386)**	0.048 **(0.038; 0.057)**	0.015 **(0.003; 0.028)**	-0.288 **(-0.407; -0.169)**	0.058 (-0.097; 0.214)	-0.317 (-0.730; 0.096)	-0.164 (-0.703; 0.373)	0.002 (-0.008; 0.014)	0.006 (-0.008; 0.022)
σ^2^	4.06	3.82	55.65	49.74	0.42	0.41	40.02	38.83	620.74	590.46	0.56	0.55
τ_00_	7.87	6.90	54.41	49.00	0.08	0.07	68.19	68.61	385.88	440.02	0.41	0.40
N	3002	2716	3002	2716	2532	2301	2606	2363	2606	2363	2997	2531
Obs.	5485	4888	5482	4885	4247	3796	4417	3940	4417	3940	5471	4391
**FOOD FREQUENCY QUESTIONARIES – FFQ**												
FS Cross-section	-0.010 (-0.027; 0.007)	-0.035 **(-0.061; -0.009)**	-0.135 **(-0.194; -0.075)**	-0.218 **(-0.305; -0.130)**	**-0.017** **(-0.022; -0.012)**	-0.013 **(-0.021; -0.005)**	0.015 (-0.044; 0.076)	-0.016 (-0.110; 0.078)	0.105 (-0.112; 0.323)	0.293 (-0.033; 0.619)	0.003 (-0.001; 0.009)	0.002 (-0.006; -0.011)
FS Longitudinal	0.322 **(0.302; 0.342)**	0.068 **(0.037; 0.098)**	1.282 **(1.214; 1.351)**	0.388 **(0.285; 0.492)**	0.070 **(0.064; 0.076)**	0.030 **(0.021; 0.040)**	-0.415 **(-0.484; -0.346)**	-0.061 (-0.172; 0.050)	-0.074 (-0.324; 0.176)	0.054 (-0.328; 0.436)	0.005 (-0.000; 0.012)	0.007 (-0.002; 0.017)
σ^2^	4.44	4.10	62.35	54.72	0.47	0.44	42.05	41.42	659.87	619.67	0.57	0.57
τ_00_	8.00	7.34	51.16	49.72	0.06	0.07	70.16	70.90	458.29	399.96	0.42	0.40
N	5538	5064	5535	5063	4786	4359	4897	4449	4897	4449	5526	5055
Obs	13,234	11,785	13,216	11,769	9877	8782	10,337	9184	10,338	9185	13,191	11,747

Random effect abbreviations: σ^2^ residual variances, τ_00_ variances of the slope, N- N-dimension of the linear predictor, Obs- observations

Model 1 (M1): crude

Model 2 (M2): adjusted for parity (number of times woman has given birth or been pregnant), mother’s education, mother’s weight before pregnancy, breastfeeding (y/n), physical activity (y/n), weight for gestational age, and energy

Globally, the cross-sectional analysis showed an inverse association between FS (/10 g) and adiposity variables (BMI – WC – HOMA-IR), and positive associations were observed in the longitudinal approach. Using information from diaries and the longitudinal approach, particularly among the adiposity variables, after adjustment, FS intake is positively and significantly associated with BMI (β = 0.044, 95%CI = 0.000; 0.088) WC (β = 0.240, 95%CI = 0.093; 0.386) and HOMA-IR (β = 0.015, 95%CI = 0.003; 0.028). Also, FS intake was inversely associated with HDL (β= -0.288, 95%CI= -0.407; -0.169) in the crude model, but it did not remain significant after adjustment. No significant associations were observed for the remaining variables.

No relevant differences were observed when analyzing FFQ with the same models and variables. Using FFQ data, there is more power, confirming the significant positive associations in the longitudinal M2 adiposity variables: BMI (β = 0.068, 95%CI = 0.037; 0.098), WC (β = 0.388, 95%CI = 0.285; 0.492), and HOMA-IR (β = 0.030, 95%CI = 0.021; 0.040). Likewise, HDL was inversely associated (β= -0.415, 95%CI= -0.484; -0.346) only in the crude model.

The sensitivity analysis only has plausible reporters (Table 4), which maintains the same tendency of the results when removing misreporters. The cross-sectional associations persist negatively. In the longitudinal diaries approach, the adjusted associations remained significantly positive for WC (β = 0.105, 95%CI 0.013; 0.198) and HOMA-IR (β = 0.010, 95%CI = 0.000; 0.021). From FFQ longitudinal approach, the associations with the same adiposity variables remain significantly positive WC (β = 0.123, 95%CI = 0.055; 0.191) and HOMA-IR (β = 0.014, 95%CI = 0.006; 0.022). Additionally, FS intake was significantly and positively associated with z-SBP (β = 0.017, 95%CI = 0.003; 0.031) after adjustment.

**Table 4:** Cross-sectional and longitudinal associations of FS (by 10 g) with cardiometabolic risk markers in plausible reporters

	BMI (kg/m^2^)		WC (cm)		HOMA- IR		HDL (mg/dL)		TG (ml)		Z-score SBP	
	M1	M2	M1	M2	M1	M2	M1	M2	M1	M2	M1	M2
**DIARIES**												
FS Cross-section	-0.046 **(-0.076; − 0.015)**	-0.007 (-0.034; 0.019)	-0.191 **(-0.294; -0.088)**	-0.057 (-0.137; 0.021)	-0.013 **(-0.022; -0.005)**	0.005 (-0.004; 0.014)	0.103 (-0.001; 0.208)	0.059 (-0.076; 0.195)	0.215 (-0.144; 0.576)	0.774 **(0.293; 1.256)**	0.010 **(0.000; 0.020)**	0.009 (-0.003; 0.022)
FS Longitudinal	0.221 **(0.186; 0.255)**	0.006 (-0.025; 0.037)	0.891 **(0.773; 1.009)**	0.105 **(0.013; 0.198)**	0.048 **(0.038; 0.057)**	0.010 **(0.000; 0.021)**	-0.288 **(-0.407; 0.169)**	0.093 (-0.062; 0.250)	-0.317 (-0.730; 0.096)	-0.156 (-0.707; 0.394)	0.002 (-0.008; 0.014)	0.006 (-0.009; 0.021)
σ^2^	4.06	1.51	55.65	15.56	0.42	0.23	40.02	34.19	620.74	543.90	0.56	0.55
τ_00_	7.87	4.56	54.41	25.38	0.08	0.11	68.19	67.27	385.88	468.40	0.41	0.38
N	3002	2534	3002	2533	2532	2160	2606	2199	2606	2199	2997	2531
Obs.	5485	4401	5482	4398	4247	3452	4417	3546	4417	3546	5471	4391
**FFQ**												
FS Cross-section	-0.010 (-0.027; 0.007)	-0.002 (-0.021; 0.016)	-0.135 **(-0.194; -0.075)**	-0.031 (-0.089; 0.025)	-0.017 **(-0.022; -0.012)**	0.002 (-0.004; 0.009)	0.015 (-0.044; 0.076)	-0.061 (-0.157; 0.033)	0.105 (-0.112; 0.323)	0.296 -0.047; 0.640)	0.003 (-0.001; 0.009)	0.006 (-0.005;0.018)
FS Longitudinal	0.322 **(0.302; 0.342)**	-0.004 (-0.026; 0.017)	1.282 **(1.214; 1.351)**	0.123 **(0.055; 0.191)**	0.070 **(0.064; 0.076)**	0.014 **(0.006; 0.022)**	-0.415 **(-0.484; -0.346)**	0.002 (-0.108; 0.114)	-0.074 (-0.324; 0.176)	0.101 (-0.296; 0.498)	0.005 (-0.000; 0.012)	0.017 **(0.003; 0.031)**
σ^2^	4.44	1.68	62.35	18.27	0.47	0.26	42.05	36.39	659.87	613.26	0.57	0.56
τ_00_	8.00	5.71	51.16	32.10	0.06	0.11	70.16	69.64	458.29	401.59	0.42	0.38
N	5538	4869	5535	4868	4786	4205	4897	4261	4897	4261	5526	3514
Obs	13,234	10,449	13,216	10,433	9877	7911	10,337	8159	10,338	8160	13,191	6226

Random effect abbreviations: σ^2^ residual variances, τ_00_ variances of the slope, N- N-dimension of the linear predictor, Obs- observations

Model 1 (M1): crude

Model 2 (M2): adjusted for parity (number of times woman has given birth or been pregnant), mother’s education, mother’s weight before pregnancy, breastfeeding (y/n), physical activity (y/n), weight for gestational age, and energy

## Discussion

This study targeted children from 4 to 13 years to estimate associations between the trajectories of FS intake and cardiometabolic risk markers, which globally show negative associations when analyzing at one point in time (cross-sectionally). However, when exploring longitudinally, positive associations were found in crude and adjusted models, particularly with the adiposity variables.

The comparison of our study is difficult since no previous studies reported the trajectories of FS and cardiometabolic risk markers with a longitudinal perspective. From the literature, previous research has mainly focused on cross-sectional approach and used as main exposure the intake of total or AS instead of FS.

A cross-sectional study from the USA [44] showed that higher AS intake was associated with an increased prevalence of metabolic syndrome in adolescents. The adjusted prevalence odds ratios of developing metabolic syndrome when comparing the participants in the second, third, fourth, and fifth quintiles versus those in the lowest quintile of AS were 2.4 (95% CI 0.6, 9.9), 5.3 (95% CI 1.4, 20.6), 9.9 (95% CI 1.9, 50.9) and 8.7(95% CI 1.4, 54.9), respectively. Another study from Japan [45] studied AS and FS intake with metabolic biomarkers in children in 8th grade finding that the intake of AS (7.6–7.9% TEI) and FS (8.4–8.8%TEI) were not significantly associated with BMI-overweight or other risks, except for AS intake ≥ 10% TEI and glucose ≥ 100 mg/dL (OR = 1.5, 95% CI = 1.04–2.19, *p* = 0.031). Also, they stated that cross-sectional studies evaluating the association with obesity are biased in an unexpected direction, and prospective cohort studies should be considered in the future. The current research with G21 results among adiposity variables (BMI – WC – HOMA-IR) confirms the positive associations when considering the longitudinal approach, both when using diaries and FFQ.

Another study in the USA examined AS with some cardiometabolic outcomes (cross-sectional in children) [46], found a significant positive association between AS and diastolic blood pressure (DBP) (β = 0.0206, *p* = 0.046), no significant association with SBP (β = 0.0126, *p* = 0.4827), positive association with TG (β = 0.109, *p* = 0.020) and no other associations with serum lipid outcomes. The authors also mentioned that longitudinal studies are needed to corroborate whether the relation between AS is causative or correlative.

Fewer studies have been done on children, although the main associations are through SSB. A Korean study [47] in children from the KoCAS cohort data found that the sugar beverage category had a positive association with TG and a negative association with HDL at baseline. After a 4-year follow-up, only SSB had a negative association with the mean arterial pressure, and no associations were found with the rest of the variables (fasting blood glucose, HDL, or TG). Additionally, a publication on Saudi [48] children/adolescents (using cross-sectional and FFQ assessment methods) found a positive correlation between WC and BMI with SSB in boys but not in girls. Also, the Framingham Heart Study in adults linked the consumption of ≥ 1 SSB per day to increased odds of developing high blood pressure [49].

A review [50] found that SSB intake has a negative effect on weight and diseases linked to obesity, mainly cardiometabolic risk. Their conclusion aligns with our hypothesis. It is crucial to consider solid foods to broaden the scope of the overall dietary sugar (especially free sugar intake). Solid foods can also be high in sugar content and have a similar lack of essential nutrients as the SSB, which is associated with poor metabolic health outcomes. Furthermore, systematic reviews found no associations among SSB consumption with blood lipids, glycemic control, and blood pressure [51] or confirmed that SSB intake promotes a higher BMI and body weight in children and adults [21].

In the current paper, using free sugar intake from all food sources in diaries, a significant positive association was observed with TG (β = 0.720, 95%CI = 0.249; 1.191) cross-sectionally; however, no significant effect was observed when using the longitudinal approach. For HDL, significant negative associations were found in the crude longitudinal models; however, after adjustment, the association did not remain significant. Also, no significant effect was observed for SBP. This might be because children, could be too young for presenting significant results in the lipid profile or blood pressure.

### Strengths and limitations

One of the study’s main strengths is using a population-based birth cohort, which grants a longitudinal approach to tracking FS intake during childhood. To our knowledge, no studies have followed FS trajectories in children in a longitudinal approach and compared with a cross-sectional approach in the same setting, which creates new knowledge. Additionally, exploring FS intake from different assessment methods -food diaries and FFQ- brings consistency among the results. Using 3-day diaries covering week/weekend days, coded and handled by trained interviewers from the area of nutrition into software developed specifically for the Portuguese population [52], could minimize the bias and support accuracy. Also, FFQ covers participants’ previous six months with various items that provide extra support for FS intake. When comparing the results from both dietary assessment methods, there is not much difference in the associations with cardiometabolic risk markers—confirming the usefulness of FFQ in large population studies. Another main strength to highlight in this paper is the use of solid and liquid foods when analyzing FS intake, filling a gap in the FS research.

One of the study’s limitations is the change in the report from parents to children. Parents reported diaries and FFQ in the first three waves, but in the last wave (13y), children reported their consumption with their parents’ help if needed. Also, another critical disadvantage to take into account is the underreporting, predominantly in children who are overweight or obese and are inclined to underestimate their intake, particularly FS sources, compared to those with a normal weight [53, 54]. As seen in a previous analysis [55], a slight drop was observed from 10 to 13 years, but after considering only plausible reporters in the analysis, the decline was no longer observed. Taking this in mind, in the current paper, a sensitive analysis was performed only in plausible reporters, and no significant differences were observed supporting our main conclusions.

FFQ could have some limitations that have been pointed out, such as broadness, vague questions, or the frequency of consumption, which could be hard to answer [56], especially for children/adolescents. Also, it could be difficult for children/adolescents to remember what they ate or how often they consumed certain foods, introducing some response bias. However, when interpreting the current results, using two dietary approaches in this analysis led to similar findings among diaries and FFQ.

## Conclusion

This study supports that longitudinal approaches are needed to obtain accurate associations between free sugar intake and cardiometabolic risk, while cross-sectional studies should be interpreted cautiously. Consequently, future research should continue to explore the longitudinal approach rather than looking at them only at one point. According to our analysis, the results are similar when using food diaries or FFQ. This also supports the use of FFQ in future studies based on a large population. Public health interventions targeting the reduction of FS intake since the early stages of life are crucial to mitigate these health risks and promote overall well-being.

## Data Availability

The data from Generation XXI are not publicly available due to privacy or ethical restrictions. The data can be made available for research proposals on request to the Generation XXI Executive Committee (generationxxi@ispup.up.pt). Further information about Generation XXI can be obtained via the Generation XXI website [www.geracao21.com] or by emailing generationxxi@ispup.up.pt.
